# The Efficacy of Blended Learning in a Pediatric Spine Deformity Management Program in Sub-Saharan Africa

**DOI:** 10.5435/JAAOSGlobal-D-22-00128

**Published:** 2023-02-03

**Authors:** Alaaeldin Azmi Ahmad, Abdallah Abushehab, François Waterkeyn, Beverly Cheserem, Massimo Balsano, Christopher Bonfield, Hamisi Shabani, Juma Magogo, Bryson Mcharo, Costansia Bureta, Fabian Sommer, Branden Medary, Ibrahim Hussain, Roger Härtl

**Affiliations:** From the Pediatric Orthopedic Surgery, Palestine Polytechnic University, Ramallah, Palestine (Dr. Ahmad); the Teaching and Research Department, An-Najah National University, Nablus, Palestine (Dr. Abushehab); the Department of Neurosciences Grand Hôpital de Charleroi, Charleroi, Belgium (Dr. Waterkeyn); the Muhimbili Orthopedic Institute, Dar es Salaam, Tanzania (Dr. Waterkeyn, Dr. Shabani, Dr. Magogo, Dr. Mcharo, and Dr. Bureta); the Department of Neurological Surgery, Weill Cornell Brain and Spine Center, New York, NY (Dr. Waterkeyn, Dr. Sommer, Mr.Medary, Dr. Hussain, and Dr. Härtl); the Aga Khan University Hospital, Nairobi, Kenya (Ms.Cheserem); the Regional Spinal Department, UOC Ortopedia A, AOUI, Verona, Italy (Dr. Balsano); and the Department of Neurological Surgery, Monroe Carell Jr. Children's Hospital, Vanderbilt University School of Medicine, Nashville, TN (Dr. Bonfield).

## Abstract

Our study assessed the efficacy of blended learning, which combines in-person learning and e-learning, in a pediatric scoliosis training program through an international collaborative effort.

**Methods:**

The course comprised two parts: the online portion, where participants reviewed educational materials for 3 weeks and met with faculty once/week for discussion, and the in-person session, where participants reviewed cases in a team-based approach and came to a consensus on treatment strategy, followed by discussion with an international expert. All participants completed a needs assessment (NA) and clinical quiz at three points: before the course, after the online session, and after the in-person session, which covered various topics in pediatric spine deformity.

**Results:**

Thirty-six surgeons enrolled in the course from 13 College of Surgeons of East, Central and Southern Africa countries. The NA assessment scores improved significantly over the course of the surveys from 67.3, to 90.9, to 94.0 (*P* = 0.02). The clinical quiz scores also improved from 9.91, to 11.9, to 12.3 (*P* = 0.002).

**Conclusion:**

The blended learning approach in a pediatric spine deformity program is effective and feasible and shows a statistically significant change in participants' confidence and knowledge base in these complex pathologies. This approach should be explored further with larger numbers and/or other spinal pathologies.

Blended learning is a relatively new form of education for surgical trainees that combines in-person learning and e-learning and has rapidly expanded in the wake of the COVID-19 pandemic. E-learning leverages internet technologies to enhance knowledge and performance. Advantages of this modality include control over content, learning sequence, and pace of learning, allowing participants to tailor their experience to meet their personal learning objectives.^1^ As standard teaching strategies may be insufficient to meet changing needs, alternatives based on direct communication and indirect information technologies are increasing in popularity.^2,3^ Blended learning not only translates theory into practice^2^ but also enables adaptive and collaborative learning and furthermore transforms the teacher's role from transmitting knowledge (instructing) to facilitating learning.^3^

With the introduction of blended learning, increasing research has focused on concerns about its effectiveness.^4^ Blended learning is highly content and context sensitive, and interdisciplinary transitions are unpredictable, so there is no guarantee that a successful blended learning application in one field will be equally successful in another.^5^ Therefore, our study aimed to assess the efficacy of blended learning in a pediatric scoliosis training program for trainees and practicing spine surgeons in sub-Saharan African nations.

## Methods

**Figure 1:**
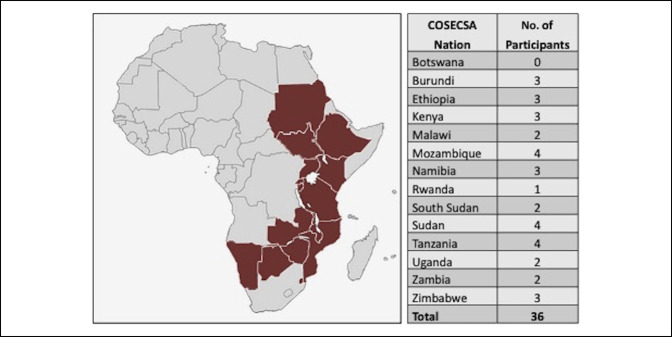
Number of participants and country of origin for the blended learning course for pediatric spinal deformity.

We conducted a prospective educational study evaluating a blended learning course for pediatric spinal deformity management. This pilot course was implemented through the Weill Cornell Global Neurosurgery Initiative^6,7,8,9^ in collaboration with the largest Surgical Training Institution in sub-Saharan Africa, the College of Surgeons of East, Central and Southern Africa (COSECSA).^10,11^ Topics covered during the course included adolescent idiopathic scoliosis, kyphosis, and early-onset scoliosis. The course involved 36 orthopaedic surgeons and neurosurgeons, of which four were residents and 32 were specialists. All participants agreed to be part of this study and have their anonymous responses to surveys collected. These individuals were nominated through 13 of the 14 COSECSA nations (Figure 1). The course comprised two parts: an online and an in-person part. Registration for the study included a mandatory needs assessment (NA) questionnaire and a knowledge-based surgical quiz before the course. Participants then completed the 3-week online portion of the course, followed by the 1-day in-person part.

### The Precourse Needs Assessment and Surgical Quiz

The NA consisted of questions about the participants' perception of their skills and knowledge in different program-related topics. The NA contained three questions for each of six topics, scored by a 10-point scale, with 1 considered “no current skills/knowledge” and 10 considered “expert skills/knowledge” and incremental gradations in between. The questions and topics were the following:

### Questions

1. Where do you see yourself now? (1 to 10)
2. Where do you want to see yourself at the end of this course? (1 to 10)
3. To what extent do you think you can use this subject in your daily practice? (1 to 10)

### Topics

4. Clinical and radiologic assessment of pediatric spine deformity
5. Nonsurgical management of pediatric spine deformity
6. Surgical management of pediatric spine deformity
7. Adolescent idiopathic scoliosis
8. Early-onset scoliosis
9. Kyphosis

The separate knowledge base surgical quiz included 15 surgical and clinical questions related to the topics mentioned above. Correct answers to these questions were not provided to assess progress on subsequent assessments.

### The Online Course

Participants were exposed to the theoretical curriculum of the course at the first stage. This part included lectures on fundamental knowledge, links to important open access papers, and audiovisual materials. One week was allowed for participants to study the basics of one topic, after which faculty members engaged the participants in a virtual discussion forum facilitated with open questions or small practical assignments. Participants, as well as the faculty, were monitored for their active participation in this part of the course by two mechanisms:

1. Faculty members were presented with information on the login times of participants (and other faculty members) so that they are aware of the amount of time participation by individuals.
2. By a discussion forum facilitated by faculty that encouraged and promoted peer discussion in the learner group.

At the end of the online course, the participants retook the same NA and quiz.

### The In-person Course

After completing the online portion of the course, the 1-day in-person case discussion began with two cases per topic. All members were divided into three groups, each group contained 12 participants. The flow of each case was as follows:

1. Presentation of the case and questions by faculty: 10 minutes
2. Discussion of the case within groups of participants: 15 minutes
3. Discussion of solutions for the case by all groups and faculty: 20 minutes

Consensus solution for each case was agreed on by the group. Then, the faculty went through the appropriate case solution in the following steps:

1. Case solution by faculty and discussion of the solution: 10 minutes
2. Reflection: 5 minutes, includes brief statements from the participants on what they have learned through the case discussion

At the end of the in-person course, the participants underwent a final NA and surgical quiz with the same questions, and the results of pre-, post-online, and post-in-person assessments and quizzes were compared.

The post-online and post-in-person NAs contained two questions for each of the six topics. There was no question number 2 (where do you want to see yourself at the end of this course?). Therefore, for proper comparing alignment, we considered removing question number 2 in the pre-online NA, so all NAs (pre-online, post-online, and post-in-person) contain 12 questions each.

### Statistical Analysis

Statistical analysis was conducted using SPSS 26.0 (IBM Corp). Continuous variables, including the results of the NA and surgical quiz, were compared using the Wilcoxon or Kruskal-Wallis nonparametric test depending on the number of samples compared. A significance threshold set to a *P* value ≤0.05.

## Results

A total of 36 participants from 13 countries joined this blended course (Figure 1); 32 (88.9%) completed the online portion, and 26 (72.2%) completed the entire course, including four residents and 22 specialists.

### The Needs Assessment Questionnaire

There was a significant improvement in the total and mean score for NA surveys (Table 1, *P* = 0.02), specifically comparing the pre-online and post-in-person assessments (Table 2). On the other hand, there was no significant difference in results between residents versus specialists participating in the course in pre-online (*P* = 0.95), post-online (*P* = 0.3), and post-in-person (*P* = 0.13) assessments.

**Table 1:** Results of the Needs Assessment Surveys

Assessment	No of Questions	Mean Score	SD	*P* value
Pre-online	12	5.6	2.3	0.02
Post-online	12	7.6	0.5	
Post-in-person	12	7.8	0.4	

**Table 2:** Comparing the Results of the Needs Assessment Surveys

Quiz	*P* value
Pre-online versus post-online	0.09
Pre-online versus post-in-person	0.009
Post-online versus post-in-person	0.1

### Surgical Quiz

There was a significant improvement in the mean quiz score throughout the course (Table 3, *P* < 0.002), especially pre-online and post-in-person quizzes (Table 4). There was no significant in the results between residents versus specialists participating in the course in pre-online (*P* = 0.52), post-online (*P* = 0.56), and post-in-person (*P* = 0.48) assessments.

**Table 3:** Results of the Surgical Quiz

Quiz	No of questions	Mean score	SD	*P* value
Pre-online	15	9.9	2.9	0.002
Post-online	15	11.9	2.7	
Post-in-person	15	12.3	2.1	

**Table 4:** Comparing the Results of the Surgical Quiz

Quiz	*P* value
Pre-online versus post-online	0.005
Pre-online versus post-in-person	0.002
Post-online versus post-in-person	0.6

## Discussion

Our study showed that a blended learning approach may improve learning in the pediatric scoliosis surgery field, particularly in low- and middle-income countries where access to surgical experts is limited. There was a significant improvement in the subjective and objective evaluation of surgical knowledge, assessed using the NA survey and surgical quiz, respectively. Several reasons are thought to be behind this favorable result. First, blended learning allows for the use of time dedicated to education more effectively. This is an issue especially in departments of surgery because of the clinical and surgical workload expected from learners, leaving limited time for education during hospital obligations. Therefore, transferring a substantial portion of time dedicated to additional learning to outside the healthcare setting is more efficient.^12^ In addition, as the online content in blended learning may be presented in any form, such as blogs, journal articles, podcasts, and videos, it was very suitable for pediatric spine surgery, which is a complex field with many possible treatment options and surgical solutions. The frequent assessment of the participant at each step in the study allowed us to track and monitor their progress and detect which step in the study had the most substantial effect on participant outcomes.

Our results are in line with other medical specialties, which have demonstrated that blended learning is an effective method for learning by participants. For example, in a study of blended learning for training maxillofacial surgeons, participants preferred online learning over the traditional alternative and were very satisfied.^13^ Blended learning was shown to be effective in reducing obstetric anal sphincter injuries in a program attended by doctors and midwives.^14^ In family planning education, blended learning resulted in the highest gains in acquiring information compared with online learning alone.^15^ Other studies have shown that blended learning has several advantages over traditional learning in other surgical fields and higher surgical qualifications can be delivered successfully with high participant satisfaction.^16^

**Figure 2:**
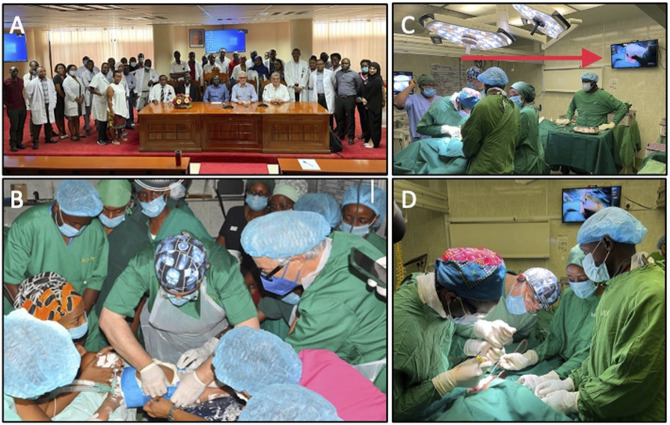
**A,** Hands-on training camp at Muhimbili Orthopaedic Institute, attended by over 30 physicians from the region and international pediatric spinal deformity experts. **B,** Instructional casting technique observed by participants for early-onset scoliosis. **C**, Intraoperative photograph with live stream of the surgery for College of Surgeons of East, Central and Southern Africa members who could not attend in person to observe. **D,** Intraoperative photograph during pediatric spinal deformity correction of international visiting surgeon teaching participant trainees.

**Figure 3:**
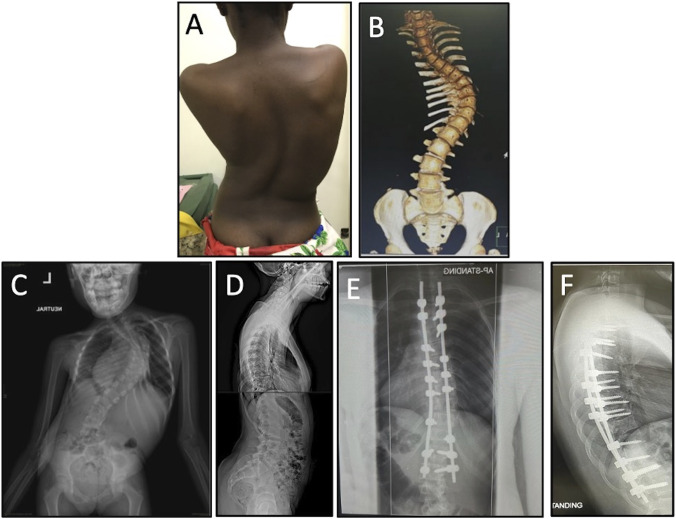
Example of a patient operated on by visiting surgeons and participants of the on-site camp at Muhimbili Orthopaedic Institute. **A,** Photograph of a patient in clinic with adolescent idiopathic scoliosis. Note the dextroscoliotic curve of the spine, unlevel shoulders, prominent shoulder blade, rib hump, and mild pelvic obliquity. **B,** Preoperative 3-dimensional reconstruction. **C,** PA radiograph demonstrating almost a 90-degree main thoracic curve with axial rotation. **D,** Preoperative lateral radiograph without any significant malalignment. Postoperative (**E**) PA and (**F**) lateral radiographs status post T3-L2 instrumented fusion with posterior column osteotomies, demonstrating deformity correction with straightening of the coronal curve and axial derotation. There were no intraoperative or postoperative complications.

One aspect of this study that was conducted but with different participants was a 1-week pediatric spinal deformity on-site surgical camp at Muhimbili Orthopaedic Institute in Dar Es Salaam, Tanzania. Because of financial and logistic constraints, most of the trainees in the blended learning course could not attend, but 30 physicians from Tanzania, Kenya, and Malawi participated (Figure 2). The course covered case conferences, casting sessions (Figure 2B), and the opportunity for trainees to observe or assist international expert visiting surgeons conduct pediatric spinal deformity correction in selected patients (Figures 2 and 3). These surgeries were also streamed live for all members of COSECSA to observe. Informed consent for the surgical procedure and use of their deidentified photographs and videos for educational purposes were obtained from all patients' families. In future iterations of blended learning applications, surgeons/trainees who were involved in the online and in-person sessions will participate in a third portion of the course where they will apply their knowledge to actual patients and in performing operations alongside expert surgeons. This experience would further solidify the principles gained during the didactic and case-based virtual portion of the course. Although logistics of organizing this type of effort are challenging, collaborative efforts such as those through the Weill Cornell Global Neurosurgery Initiative and COSECSA are such platforms to achieve this.

## Limitations

It should be noted that although we did demonstrate statistically significant differences over the course of the study, this does not necessarily apply any causation or causality. The number of participants decreased from the time of enrollment to the end of the course. Although this is a significant limitation, it is not unique to our course and study. This is one of the major issues associated with online learning in general.^17^ Furthermore, the number of participants is relatively small, and a larger sample size with more committed members is needed to have a more comprehensive and complete view of the efficacy of blended learning.

## Conclusion

The blended learning approach for pediatric spinal deformity is effective and feasible and shows a statistically significant improvement in participant’s performance. Larger studies are needed for further assessment of this topic.

## References

[1] Ruiz JG Mintzer MJ Leipzig RM: The impact of E-learning in medical education. Acad Med 2006;81:207-212. 16501260 10.1097/00001888-200603000-00002

[2] Keifenheim KE Velten-Schurian K Fahse B : A change would do you good": Training medical students in Motivational Interviewing using a blended-learning approach - a pilot evaluation. Patient Educ Couns 2019;102:663-669. 30448043 10.1016/j.pec.2018.10.027

[3] Gray K Tobin J: Introducing an online community into a clinical education setting: A pilot study of student and staff engagement and outcomes using blended learning. BMC Med Educ 2010;10:6. 20100354 10.1186/1472-6920-10-6 PMC2828452

[4] Liu Q Peng W Zhang F Hu R Li Y Yan W: The effectiveness of blended learning in health professions: Systematic review and meta-analysis. J Med Internet Res 2016;18:e2. 26729058 10.2196/jmir.4807 PMC4717286

[5] Kassab SE Al-Shafei AI Salem AH Otoom S: Relationships between the quality of blended learning experience, self-regulated learning, and academic achievement of medical students: A path analysis. Adv Med Educ Pract 2015;6:27-34. 25610011 10.2147/AMEP.S75830 PMC4293215

[6] Coburger J Leng LZ Rubin DG : Multi-institutional neurosurgical training initiative at a tertiary referral center in mwanza, Tanzania: Where we are after 2 years. World Neurosurg 2014;82:e1-e8. 23023049 10.1016/j.wneu.2012.09.019

[7] Hartl R Ellegala DB: Neurosurgery and global health: Going far and fast, together. World Neurosurg 2010;73:259-260. 20849773 10.1016/j.wneu.2010.02.047

[8] Schmidt FA Kirnaz S Wipplinger C Kuzan-Fischer CM Hartl R Hoffman C: Review of the highlights from the first annual global neurosurgery 2019: A practical symposium. World Neurosurg 2020;137:46-54. 31996336 10.1016/j.wneu.2020.01.140

[9] Wait SD Hartl R: Multi-institutional American team teaches neurosurgery in underserved Tanzania. World Neurosurg 2010;73:610-611. 20934137 10.1016/j.wneu.2010.06.043

[10] Freitas DM Munthali J Musowoya J : Surgical registrars' perceptions of surgical training and capacity in Zambia: Results from three COSECSA affiliated training hospitals. Am J Surg 2018;215:744-751. 28764850 10.1016/j.amjsurg.2017.07.023

[11] Kahamba JF Assey AB Dempsey RJ Qureshi MM Hartl R: The second african federation of neurological surgeons course in the East, central, and southern Africa region held in dar es Salaam, Tanzania, january 2011. World Neurosurg 2013;80:255-259. 22120325 10.1016/j.wneu.2011.07.021

[12] Senkoylu A Senkoylu B Budakoglu I Coskun O Acaroglu E: Blended learning is a feasible and effective tool for basic pediatric spinal deformity training. Glob Spine J 2021;11:219-223. 10.1177/2192568220916502 PMC7882832 32875908

[13] Ali-Masri H Hassan S Fosse E : Impact of electronic and blended learning programs for manual perineal support on incidence of obstetric anal sphincter injuries: A prospective interventional study. BMC Med Educ 2018;18:258. 30419884 10.1186/s12909-018-1363-3 PMC6233260

[14] Munro V Morello A Oster C : E-Learning for self-management support: Introducing blended learning for graduate students - a cohort study. BMC Med Educ 2018;18:219. 30249238 10.1186/s12909-018-1328-6 PMC6154791

[15] Gunzburg R Szpalski M Lamartina C: Postgraduate education in spine surgery: The blended online learning concept. Eur Spine J 2018;27:2059-2061. 30084013 10.1007/s00586-018-5715-9

[16] Thomas P Kern D Hughes M Chen B: Curriculum Development for Medical Education: A Six-Step Approach, 3 edn. Baltimore, MD, Johns Hopkins University Press, 2016.

[17] Aryal KR Pereira J: E learning in surgery. Indian J Surg 2014;76:487-493. 25614725 10.1007/s12262-014-1092-8 PMC4297999

